# Qualitative evaluation of mental health services for clients with limited English proficiency

**DOI:** 10.1186/1752-4458-7-27

**Published:** 2013-12-02

**Authors:** Sita G Patel, William M Firmender, Lonnie R Snowden

**Affiliations:** 1 Palo Alto University, 1791 Arastradero Road, Palo Alto, CA 94304, USA; 2 University of California at Berkeley, School of Public Health, 235 University Hall, Berkeley, CA 94720, USA

**Keywords:** Limited English proficiency, Threshold Language Policy, Language interpreters, Access to mental health services, Vietnamese Americans, Latino Americans

## Abstract

**Background:**

To meet federal requirements under Title VI of the Civil Rights Act, the state of California instituted policies requiring that comprehensive mental health services in native languages be made available to limited English proficiency (LEP) populations when concentrations exceed “threshold” levels.

**Methods:**

This paper builds on promising results from quantitative evaluations by reporting on qualitative interviews with Latino and Vietnamese LEP clients in mental health services (N = 20) to examine the awareness, impact, and implications of these threshold language policies.

**Results:**

Results suggest that, while individuals are often not aware of the policies themselves, the language-related services they receive that are prompted by the policies are critical to treatment initiation and retention. Results also convey the complexities of using interpreters for sensitive psychological topics, and suggest that, for LEP individuals seeking mental health treatment, providers who speak their native languages are generally preferred.

**Conclusions:**

Access to language-appropriate services seems to be an important part of why LEP populations seek mental health treatment. However, there are multiple variables that factor into the usage and usefulness of such services.

## Background

### Healthcare disparities for limited English proficiency individuals

In 2008, over 34 million United States residents spoke Spanish at home (an increase of 20% since 2000), and over 8 million spoke Asian or Pacific Island languages at home (an increase of 18% since 2000) [1,2]. Due to limited English proficiency (LEP), many of these immigrants to the United States are at a disadvantage when seeking treatment for physical and psychological health problems [3-5]. Communication challenges lead to limited access to appropriate treatment, and lower retention once engaged in treatment [6]. These structural barriers to care may be particularly salient in mental health settings, where there are fewer non-verbal tests to assess for illness and good diagnosis often depends on clear, accurate descriptions of the symptoms [7]. Given the rising LEP population and these significant treatment challenges, it is imperative that we develop ways to reduce health care disparities among LEP individuals.

Title VI of the U.S. Civil Rights Act requires that no one be denied services on “national origins” grounds, and for courts and the U.S. Office of Civil Rights, this prohibits neglecting language assistance needs of LEP persons [8]. Several states have adopted supplemental legislation [9]. The Department of Health and Human Services issued “Cultural and Linguistic Standards” (CLAS), providing guidance for compliance with Title VI obligations. The CLAS stipulate, among other requirements, that language assistance services be provided at all points of contact, that consumers be notified that such services are available, that competence of translation services be assessed, and that family and friends not be used as translators [10]. However, public awareness that legal obligations make language assistance services mandatory remains limited: in a large, representative sample of LEP persons, only 37% reported any knowledge of language assistance laws [11].

In the state of California, over 28% of the population speaks Spanish at home (compared with 12% nationally) and almost 10% of the population speaks an Asian or Pacific Island language at home (compared with less than 3% nationally) [1,2]. California adopted a “threshold language policy” in response to Title VI and other obligations [9]. The policy requires that once a given county’s foreign language population reaches 5% of the total population, language services are required [12]. Quantitative evaluation of these policies has been promising: Asian-language LEP individuals are using mental health services at a greater rate than before the policies were enacted [12,13]. The quantitative data presented in these studies are encouraging, and yet are removed from the lived experience of LEP individuals seeking assistance.

### Barriers and facilitators to treatment

Research confirms that cultural and language-related factors prevent LEP individuals from seeking treatment. Both Latino and Asian older adult immigrants with LEP are less likely to utilize health services, when compared to their peers who were proficient or fluent in English [5]. This suggests that language fluency, above and beyond cultural factors, plays a role in the gaps in treatment-seeking behavior, placing LEP individuals at greater risk for poor psychological and physical health. Research comparing service use among Latino and Asian immigrant adults with and without English proficiency found that language-proficient immigrants with psychiatric disorders were more likely to seek mental health services when they had multiple diagnoses, or if they rated their own mental health as poor. In contrast, no such factors predicted mental health service use among Asian immigrants with LEP [6]. Interestingly, Asian LEP individuals are less likely than Latino LEP individuals to use any mental health services at all - with 22.1% of the Latino LEP sample using some type of mental health service, while only 13.8% of the Asian LEP population reported service use [6]. This preliminary research suggests that language, above and beyond culture, may play a role in preventing treatment. However, it remains unclear whether, given the opportunity to engage in treatment in one’s native language, LEP individuals would view and engage with mental health services differently.

### Complex perspectives on interpreters

Interpreters are the most common medium for providing language assistance, and research on the role of interpreters presents a mixed picture in terms of the quality, utility, role, and benefit of interpreters for LEP individuals receiving health care. A quantitative study with Spanish-speakers exploring how interpreters can be both a facilitator and a barrier to effective treatment found that patients and physicians occasionally disagreed on what makes a good interpreter. Patients primarily wanted their interpreters to be accurate, helpful after they have left the doctor’s office, available, and capable of keeping the information confidential. The characteristics of interpreters that patients and physicians significantly disagreed on were “helpful after doctor’s visit” and “personal familiarity”, as well as whether the interpreter should be the same gender as the patient [14]. Spanish speaking patients did not mind having their friends or family members present to translate for them, reporting an 85.1% satisfaction rate. In contrast, physicians only reported a 62% satisfaction rate when family members or friends served as translators [14]. This study underscores the importance of a more nuanced, in depth, qualitative look at patients’ perspectives on interpreters.

One qualitative study did explore factors viewed to increase quality of care among Chinese and Vietnamese LEP individuals. The authors found that, in contrast to Latino LEP individuals, Asian American patients did not feel comfortable with their friends or family members serving as interpreters at the doctor’s office – citing that this role alters the family dynamic. Patients also worried that what they were saying to the doctor was not being fully or accurately translated. In keeping with findings for Latino LEP individuals, Asian Americans preferred interpreters who were the same gender as themselves [15]. Patients in this study also reported confusion about how to acquire services in the United States medical system, especially when seeking urgent care. They often had difficulty accessing the appropriate people by phone due to the language barrier [15]. Furthermore, the extent to which clients trust an interpreter is an important factor when discussing indirect client to clinician communication [16]. Two main factors stand out for clients when it comes to interpreters: personal qualities, such as empathy and respectfulness, as well as continuity of care between the client and the interpreters. These findings warrant further study – in terms of how interpreters are viewed by patients, patients’ preferences and reservations with different interpreter roles, and how mental health care systems can best connect with LEP individuals in treatment settings.

### The present study

We lack in-depth knowledge from LEP service recipients’ points of view as to what is more or less helpful about the language assistance services they receive and, in general, how they experience language assisted mental health service provision. Beginning to fill this void, we conducted focused, qualitative interviews with LEP individuals receiving mental health treatment in public clinics. We included individuals who speak two of California’s most widely-spoken non-English languages, Spanish and Vietnamese. We probed several areas of required threshold language programming to understand respondents’ experiences in several areas of concern: awareness that threshold language policy compelled service provision in Spanish or Vietnamese and awareness that language assistance was available; and respondents’ experience with translated written materials, interpreter services, and 24 hour crisis line. Because of the very limited research on limited English proficiency issues, this article contributions to an important field of research for which very little is known.

## Methods

### Recruitment

First, researchers contacted the directors of four mental health clinics in California’s San Francisco Bay Area, who each agreed to allow researchers to recruit participants at their clinics. Second, clinicians at each clinic were contacted and given informational flyers (translated into Spanish and Vietnamese), which they distributed to their LEP clients. Flyers were also left in clinic waiting rooms. Clients who were interested in participating called the phone number indicated on the recruitment flyer to schedule an individual interview.

### Participants

Participants (N = 20) in this study included clients with limited English proficiency whose primary languages were Vietnamese and Spanish. Of the 10 Vietnamese participants, all were female. The average age was 50.6, ranging from 33 to 69 years, and the average length of time in the U.S. was 23.8 years, ranging from 13 to 29 years. Of the 10 Latino (Spanish-speaking) participants, 7 were female. The average age was 42.3, ranging from 22 to 61 years, and the average length of time in the U.S. was 18.6 years, ranging from 6 to 44 years. Most participants were unemployed, with minimal elementary-level education. All participants were actively engaged in mental health services. Participants self-reported their psychiatric problems during the interview. While some identified a specific diagnosis, others described symptoms, which researchers coded into broad categories of psychiatric symptoms according to DSM-IV-TR diagnostic criteria. Among the Latino sample, depressive symptoms were the most commonly cited problems (n = 7), followed by relationship issues and domestic violence (n = 3 each). Among the Vietnamese sample, depression and psychotic symptoms were most common (n = 5 each). Additional characteristics of the sample are shown in Table 1.

**Table 1:** Demographic Characteristics

***Variable***	***Latino sample***	***Vietnamese sample***
Female gender	7	10
**Education**		
Less than 8^th^ grade	8	3
High school/GED	1	2
Some high school	0	1
Some college	1	3
College degree	0	1
**Relationship status**		
Single	1	4
Married	4	1
Separated	4	1
Divorced	1	2
Widowed	0	1
Unknown	0	1
**Employment status**		
Employed	2	1
Unemployed	8	8
Student/in training	0	1
**Psychiatric diagnosis/symptoms**		
Depression	7	3
Anxiety	1	2
Domestic violence	3	0
Trauma	2	1
General medical condition/somatic	2	1
Substance use	2	0
Schizophrenia	1	5
Relationship problems	2	0
Insomnia	1	3
Panic	1	0

### Procedures

The Institutional Review Board of the University of California, Berkeley approved the study procedures as the qualitative arm of a larger quantitative study evaluating California threshold language policies [12,13]. The first author conducted the Spanish-language interviews, and two additional field interviewers conducted the Vietnamese-language interviews. Informed consent was obtained prior to participation, and each participant received $20 for their participation in the study. Using a semi-structured interview format, participants were first asked about demographic, diagnostic, and treatment histories. They were then asked a series of questions about their awareness and use of LEP-related services.

The LEP-portion of the interview began with the following introductory statement: *For people who are not comfortable with English*, *there are certain things that clinics like this have to do to make it easier for those people to come here*. Then, interviewers asked participants if they were aware of certain services offered through the language threshold policies, if they used the services, and if the services were helpful. Items discussed, in terms of awareness, use, and helpfulness, included: 1) *This clinic tells people in the community that the clinic is here to help them if they need it*. 2) *This clinic has to translate things presented in writing into your language*. 3) *This clinic has to make sure that there are people here who speak your language so that you know everything that will happen*. 4) *There is a phone line with people to speak to in your language that people can call 24 hours a day*. Interviews lasted an average of 45 minutes, were audiotaped, transcribed *verbatim* in Spanish and Vietnamese, and translated into English by two external translators – one for the Spanish interviews and one for the Vietnamese interviews. The English transcriptions were then independently coded by the first author and one of three project assistants using nVivo qualitative data analysis software (QSR International Pty Ltd. Version 9, 2011). To increase validity, any coding discrepancies were evaluated through discussion among the researchers before a final coding decision was made. All statements relevant to threshold language policies and barriers/facilitators to treatment initiation and participation were identified.

## Results

### Language policy awareness

Many, but not all, participants were unaware of the statewide language policies regarding access to mental health services. For example, when asked about specific clinic policies, participants responded:

“Nobody hears about [the 24-hour help line].”

“I did not know that. I only knew that there were people who helped….I knew that there were people willing to help, but I did not know that there were so many Vietnamese documents.”

“I know about all these laws.”

“I realized [that LEP policies existed] when they brought me here and told me.”

### Language policies as a facilitator to treatment initiation and retention

For about three quarters of participants, access to services in their native language was a facilitator to *starting* treatment. For example:

“That they speak Spanish [made it easier to seek help]. If they spoke English who knows if I would have come, because how would I understand?”

“If I encountered any difficulty, I immediately went to see a Vietnamese doctor to speak with him.”

“[LEP services are] very useful because they give me my therapy in Spanish and all kinds of information in Spanish.”

“…a social worker who spoke Spanish called me. So it was not difficult, the psychiatrist also speaks Spanish and that is why it was not so difficult.”

Having a health care professional who spoke participants’ native language also helped them *stay* in treatment, in part because they felt comfortable receiving the care in their native language, felt able to be open about their problems, and felt overall more understood than they would with an English-speaking provider. For example:

“I am telling you that it is a blessing from God that there are [LEP services]…they are very, very useful. When we speak in our own language, it becomes easier for us to explain to the doctor or the person helping us what is going on with us, what it is that we have and how we can help ourselves.”

“…[health care providers] speaking in my own language means understanding 100% of my needs…it feels like they are understanding everything that I am saying, because I will confide in them more.”

“…it was easier to contact, to speak with a Vietnamese need Vietnamese doctors, Chinese need Chinese doctors, Americans need American doctors”

Several respondents underscored the importance of *mental health* treatment in particular being in their native language, because of the unique cognitive and emotional issues found in many mental illnesses. For example:

“I want to have Vietnamese counselors, Vietnamese doctors…because…when my mind was gone, my English was gone too, so I could not explain what I want to those who do not understand Vietnamese. With a Vietnamese doctor, it was easier to contact and to speak with them [when I was ill].”

“Because I was sick…my English language was not fluent, so I needed people to translate.”

“Can you imagine if there wasn’t someone who spoke Spanish, who was able to understand me, who I could tell what I feel when I am in pain and how I feel?”

### Interpreters

About three quarters of participants reported using interpreters and finding them to be useful. For example:

“In the beginning with the psychiatrist it was a little difficult because he didn’t speak much Spanish, and I didn’t speak much English. So they gave me an interpreter and that helped me…I understood well what they were saying and it was perfect for me that there are those kinds of services.”

“It helps to have a translator.”

“Not understanding English was difficult, but sometimes there were case workers who translated, so it was not difficult.”

“Without interpreters, I didn’t know how to speak with the doctor.”

“There was Ms. H., a Vietnamese social worker who did the translation, so I did not worry…it was convenient…it was not that difficult. It helps me continue to see the doctor…it’s convenient to see the doctor.”

“The interpreter helps me understand a lot.”

However, about half the participants in the sample also expressed reservations about using interpreters. For example:

“No I do not like to have an interpreter because they do not say it the way I want them to. I understand a lot of English and I have had interpreters who I would just end up saying ‘please do not interpret for me anymore. You are not saying it the way I want you to.’”

“The first doctor spoke English, and Mr. H. translated. With the other doctor, I spoke Spanish and it was a lot easier…it is never the same if there is a translator because it is not as direct.”

“Wherever I went without a Vietnamese [provider], I worried a little bit. At some Chinese hospitals there is someone who does a little bit of translation…but really there should be Vietnamese staff.”

“[Using an interpreter] was more difficult than with Vietnamese doctors. The most helpful was if I met with Vietnamese doctors, they gave me instructions or spoke Vietnamese, and I could understand better. If I met with American doctors, they also can help Vietnamese [people], but due to a language difference, it would be a little bit more difficult.”

“When reading English, I could not understand, so I had to show the papers to the case worker and the case worker had to spend more time [with me]. It would be very time consuming if there were no Vietnamese documents. With Vietnamese documents, we could bring them home, read, and understand slowly.”

Again, several participants highlighted issues with interpreters that are particularly salient in a mental health care setting. For example:

“Sometimes I wanted to talk about womanly things but [the interpreter] was there and I was embarrassed…When there were times when I had relationship issues with my husband…I would just talk about what was necessary.”

“I do not like having an interpreter because I feel like there are things that I do not want to say because there is a third person there.”

“I know a lady who needs to talk with a psychologist, and she feels uncomfortable because she needs an interpreter. My son was also with a psychologist who spoke English, and he decided to get out [of treatment] because he said that [the psychologist] didn’t understand much, maybe because of the language or because of the culture.”

## Discussion

The present article adds to our understanding of California’s threshold language policies in several significant ways. Results suggest that, despite policies requiring notification, many LEP individuals in this sample remained unaware of California policies requiring language services. Despite their participation in psychiatric care, respondents proved even less aware than LEP persons at large [11] that legal requirements compelled treatment programs to meet their language assistance needs. Despite this relative lack of awareness, participants used and very much appreciated language services as part of their mental health care. Nevertheless, many participants indicated a preference for mental health care providers who speak their native language, and expressed reservations about treatment with interpreters.

Indeed, the single factor that contributed most to LEP individuals’ use of mental health services was access to providers who spoke their native languages. Many participants reported reservations about using interpreters and a discomfort with their mental health information being filtered through another individual. They also reported feeling a strong cultural and linguistic connection to providers when communication was direct and in their native languages. While research on medical services in general point to the value of direct patient communication [17], it may be that providers speaking their patients’ native languages is particularly critical for mental health services. In a review of the literature on interpreter service use in psychiatric settings, the use of professional interpreters was found to improve patient disclosure and satisfaction [18]. However, this conclusion compared psychiatric evaluation with an interpreter to evaluation done in English. What emerges from the present article is the importance of going beyond interpreter use, to increasing the number of service providers who can directly provide mental health services in patients’ native languages. Because individuals suffering from mental illnesses may experience cognitive deficits and particularly sensitive emotional struggles, challenges to communication are often exacerbated and direct communication is even more imperative to effective evaluation and treatment.

A large body of research underscores the importance of the therapeutic alliance between providers of mental health services and their patients [16,19-21], and this alliance may be hindered when a third person is present and actively engaging in the dyadic process of psychotherapy [21]. In contrast to patients seeking medical care for physical illness, individuals seeking mental health care are asked to discuss information that may feel particularly sensitive and private, such as emotional or relationship issues. This kind of sensitive personal information may be most effectively communicated directly with one’s provider, rather than being filtered through a third party interpreter. Additionally, there exists the possibility that an interpreter may not accurately convey exactly what the patient intends to say, which may lead to a rupture in the therapeutic alliance if the patient does not feel as if he or she is being heard [15]. Even the most skilled and highly trained interpreters can make mistakes, and this problem can become exacerbated when the interpreter is a family friend or an ad hoc interpreter [22].

Another potential problem with the use of translators is that of confidentiality within a small community where many of the community members are familiar with one another. Research demonstrates that those within closely connected communities may be afraid of having others within their community hear about the problems they are experiencing [23,24]. Since mental health is often a particularly stigmatizing topic, it is likely that this issue may be particularly salient in community mental health settings [9]. In immigrant communities, this issue of confidentiality may arise when the only available translators are members of the same community. LEP individuals should receive treatment that meets their psychological needs and also satisfies any concerns about confidentiality. If they are worried about confidentiality within their community, they should be given the choice to see a different professional with whom they feel more comfortable.

## Limitations

Several limitations are important to note. First, participants in this study include a small sample and are not representative of all LEP individuals (e.g., 9 out of 10 Latino participants were Mexican, and Vietnamese participants were all female). Participants lived in either urban or suburban settings and so results may not generalize to rural or other kinds of settings. Second, all participants in this study were actively engaged in treatment, and so results do not capture those LEP individuals who start and then terminate treatment, or who need but do not initiate treatment. Third, coding qualitative data is in its nature subjective, so results should be interpreted with caution.

## Conclusion

The present study suggests that California’s threshold language services may be integral to LEP individuals’ initiating and staying in mental health treatment. Access to interpreters, emergency phone lines, written materials and providers all made help-seeking easier for LEP participants. Results also indicate that LEP individuals appreciate and have reservations about interpretive services as part of their mental health treatment. Therefore, we urgently need research that will clarify not only quality of interpretation, but will also elucidate how interpreters function as social actors and with what consequences for client behavior.

## Competing interests

The authors declare no competing interests.

## Authors’ contributions

SP collected and analyzed the qualitative data, formulated the research questions and manuscript structure, wrote the initial draft of the manuscript, and edited the final revisions. WF analyzed the data, conducted literature review, and helped draft and the manuscript. LS is the PI on the parent study, received the NIH-funded grant in support of the research, and reviewed, co-authored, edited, this manuscript. All authors read and approved the final manuscript.
